# To William Proffit, a Brazilian farewell

**DOI:** 10.1590/2177-6709.23.6.011-012.edt

**Published:** 2018

**Authors:** Flávia Artese, Marco Antonio de Oliveira Almeida

**Affiliations:** 1 Universidade do Estado do Rio de Janeiro, Departamento de Odontologia Preventiva e Comunitária (Rio de Janeiro/RJ, Brazil).; 2 Pós-Doutor em Ciências da Saúde, University of North Carolina System (Chapel Hill/NC, EUA). Livre-docente, Universidade do Estado do Rio de Janeiro (Rio de Janeiro/RJ, Brazil).

Since its first edition in 1996, the DPJO has been publishing an interview with professionals who have significantly contributed to our area. Our purpose is to create a permanent record of these personalities. The interview with Dr. William Proffit began its preparation in July, but unfortunately the questions that were carefully prepared by our interviewers were never answered. On the morning of September 30th, 2018, Dr. William Proffit passed away and the orthodontic world was in mourning. 

A number of obituaries were written in many journals around the world, bidding farewell and most specially describing the important legacy that Dr. Proffit left us. Professor William Proffit accepted the position as professor and chair of the Orthodontic Department of the University of North Carolina in 1975, where he stayed for 26 years. During this period he took part in the education of countless orthodontists, some of these already prestigious educators from different parts of the world. Among these we may include Marco Antonio Almeida, Professor of Orthodontics of the Rio de Janeiro State University, who spent two years with Dr. Proffit at the University of North Carolina. In this opportunity they created strong bonds and he was therefore invited to organize the unfinished interview. Thus, we wrote this editorial together. 

Very few professionals reached such resourcefulness in their profession and became famous worldwide and recognized by most as the best of the world. We can name Pelé in soccer, Mohamed Ali in boxing, and with no doubt, William Proffit in Orthodontics. There is no orthodontist who does not recognize his name, or has not listened to one of his lectures or has not read one of his books. However, since the roots of our journal are Brazilian, we would like to recall some important impressions that Professor Proffit left in Brazil. 

Each course presented by Dr. Proffit left teachings for a lifetime. His capacity of transforming information into something simple to understand was notable. His first course in Rio de Janeiro, in the beginning of the 80’s, left the participants marvelled with Orthognatic Surgery. He changed all that was done in this subject at that time, a result of a lifetime of dedication to research on surgical treatment. This knowledge and experience summed up in a textbook on this topic, written together with Drs. Bell and White, and later on, in the well known hierarchy of stability of surgical procedures from the University of North Carolina^1^.

He returned to Rio de Janeiro again in the late 80’s, and this time we had contact for the first time with the primary failure of eruption^2^, a clinical situation that had already been seen in our practices, but we mistakenly diagnosed it as multiple ankylosis. But, we believe that his highlight in Brazil was in 1998. Once again in the city of Rio de Janeiro, at the Hotel Gloria, when he gathered 1,000 orthodontists, the auditorium’s maximum occupancy, in a 12 hour course. It was really a landmark in the history of Brazilian orthodontics. Everyone wanted to meet Proffit personally. After all, his textbook “Contemporary Orthodontics” had just been translated into Portuguese for the first time, and published in 1995, under the supervision of Professor Nelson Mucha. 

In this same book, we had the chance to read the chapter on Etiology of Malocclusions, where the paper “Equilibrium Theory Revisited”^3^ was cited. In our opinion, a mandatory reading for all dentists. So important was this paper that Kevin O’Brien mentioned it in his tribute to Proffit. This paper is significant for those who knew him and understood his thoughts on the influence of function in malocclusions. Certainly this way of reasoning gave rise to some questions in the aforementioned interview, which will unfortunately remain unanswered with a most current perspective, such as: *Should myofunctional therapy be indicated? Before, during, or after orthodontic treatment?* Something to think about...

But for someone who influenced countless professionals, taught so many, and lived through so many changes along his brilliant career, maybe our biggest curiosity would be the answer to these questions: *What are the most significant factors that will affect the practice of orthodontics for the next generation, and what advice(s) would you give to young enthusiastic orthodontists at the early days of their careers?*

We will certainly find good answers in the lines and maybe in the small print of the legacy that he left to Orthodontics in his work. Especially through the respect which he placed in his profession during all his life. And in his example of generosity, after all the knowledge that he has left us will be eternal. To Dr. William Proffit our profound gratitude and the assurance that he will be greatly missed.
